# Combined Metabolic Activators Decrease Liver Steatosis by Activating Mitochondrial Metabolism in Hamsters Fed with a High-Fat Diet

**DOI:** 10.3390/biomedicines9101440

**Published:** 2021-10-11

**Authors:** Hong Yang, Jordi Mayneris-Perxachs, Noemí Boqué, Josep M. del Bas, Lluís Arola, Meng Yuan, Hasan Türkez, Mathias Uhlén, Jan Borén, Cheng Zhang, Adil Mardinoglu, Antoni Caimari

**Affiliations:** 1 Science for Life Laboratory, KTH Royal Institute of Technology, SE-17165 Stockholm, Sweden; hong.yang@scilifelab.se (H.Y.); meng.yuan@scilifelab.se (M.Y.); mathias.uhlen@scilifelab.se (M.U.); cheng.zhang@scilifelab.se (C.Z.); 2 Department of Diabetes, Endocrinology and Nutrition, Girona Biomedical Research Institute (IDIBGI), Hospital Universitari de Girona Doctor Josep Trueta, 17190 Girona, Spain; jmayneris@idibgi.org; 3 Center for Pathophysiology of Obesity and Nutrition (CIBEROBN), Instituto de Salud Carlos III, 28029 Madrid, Spain; 4 Eurecat, Centre Tecnològic de Catalunya, Technological Unit of Nutrition and Health, 43204 Reus, Spain; noemi.boque@eurecat.org (N.B.); josep.delbas@eurecat.org (J.M.d.B.); lluis.arola@urv.cat (L.A.); 5 Nutrigenomics Research Group, Department of Biochemistry and Biotechnology, Campus Sescelades, Universitat Rovira i Virgili, 43007 Tarragona, Spain; 6 Department of Medical Biology, Faculty of Medicine, Atatürk University, Erzurum 25030, Turkey; hturkez@atauni.edu.tr; 7 Department of Molecular and Clinical Medicine, University of Gothenburg and Sahlgrenska University Hospital, SE-40233 Gothenburg, Sweden; Jan.Boren@wlab.gu.se; 8 School of Pharmaceutical Sciences, Zhengzhou University, Zhengzhou 450001, China; 9 Centre for Host-Microbiome Interactions, Faculty of Dentistry, Oral & Craniofacial Sciences, King’s College London, London WC2R 2LS, UK

**Keywords:** NAFLD, combined metabolic activators, transcriptomics, mitochondrial metabolism

## Abstract

Although the prevalence of non-alcoholic fatty liver disease (NAFLD) continues to increase, there is no effective treatment approved for this condition. We previously showed, in high-fat diet (HFD)-fed mice, that the supplementation of combined metabolic activators (CMA), including nicotinamide riboside (NAD^+^ precursor) and the potent glutathione precursors serine and N-acetyl-l-cysteine (NAC), significantly decreased fatty liver by promoting fat oxidation in mitochondria. Afterwards, in a one-day proof-of-concept human supplementation study, we observed that this CMA, including also L-carnitine tartrate (LCT), resulted in increased fatty acid oxidation and de novo glutathione synthesis. However, the underlying molecular mechanisms associated with supplementation of CMA have not been fully elucidated. Here, we demonstrated in hamsters that the chronic supplementation of this CMA (changing serine for betaine) at two doses significantly decreased hepatic steatosis. We further generated liver transcriptomics data and integrated these data using a liver-specific genome-scale metabolic model of liver tissue. We systemically determined the molecular changes after the supplementation of CMA and found that it activates mitochondria in the liver tissue by modulating global lipid, amino acid, antioxidant and folate metabolism. Our findings provide extra evidence about the beneficial effects of a treatment based on this CMA against NAFLD.

## 1. Introduction

Hepatic steatosis (HS) is defined as the excessive accumulation of fat in the liver (>5.5% tissue weight), and it is the characteristic feature of non-alcoholic fatty liver disease (NAFLD) [1]. NAFLD is a widespread metabolic disorder that can be considered a multifactorial disease and that refers to a group of conditions including HS and various degrees of liver inflammation, such as non-alcoholic steatohepatitis (NASH). NAFLD can progress to cirrhosis, and ultimately hepatocellular carcinoma (HCC), which are much more severe liver diseases [1,2,3]. It has been reported that the global prevalence of NAFLD is approximately at ~25% [1,4,5]. Although research in drug development for NAFLD is intense and advancing rapidly, there are still significant unmet challenges with no effective drug approved for this condition [4]. Therefore, it is of paramount importance to find effective treatments against this disease and to elucidate the underlying mechanisms.

So far, the most effective strategy to counteract NAFLD is weight loss and the adherence to healthier life style habits, including hypocaloric diets and physical exercise [5,6,7]. However, this long-term strategy fails in most of the subjects because it is extremely difficult to maintain body weight reduction (lost weight is usually gradually regained) and due to the poor adherence to hypocaloric diets, which, in many cases, have a low palatability. In a review published some years ago, members of our team suggested the adherence to a highly palatable Mediterranean diet and the use of multi-ingredient-based supplements that act against complementary targets (such as vitamin E and silybin, PUFAs and vitamins, and synbiotics) as potential strategies to tackle effectively this multifactorial disease [5]. In this line, other members of this current team proposed the use of a multi-ingredient supplement including serine (glutathione (GSH) precursor), N-acetyl-l-cysteine (NAC, GSH precursor), nicotinamide riboside (NR, NAD^+^ precursor), and L-carnitine (which promotes the uptake of fat to mitochondria) to boost fatty acid oxidation and to counteract the insufficient capacity to remove incomplete products of fatty acid oxidation, which is one of NAFLD’s main hallmarks [8,9]. Oxidative stress has a significant role in NAFLD’s pathogenesis and is characterized by impaired function of electron transport chains, impaired oxidation and increased production of reactive oxygen species (ROS) [10,11,12]. GSH is the most abundant endogenous antioxidant in response to oxidative stress and increased ROS [13,14,15]. Therefore, we hypothesized that a three-step strategy including (i) activating mitochondrial fatty acid uptake, (ii) increasing fatty acid oxidation, and (iii) increasing the availability of GSH could be employed to decrease HS in NAFLD patients [5,9,16,17,18].

To validate our hypothesis, we firstly performed a study in which a multi-ingredient supplement based on combined metabolic activators (CMA) including serine, NR and NAC was given to mice fed a high-fat diet (HFD) for 14 days. The results showed that supplementation of CMA significantly decreased the amount of liver fat by promoting the fat oxidation in mitochondria in the liver [9,19]. We further studied the effect of the CMA including the aforementioned metabolic activators but adding also L-carnitine tartrate (LCT, salt form of L-carnitine) in a one-day proof-of-concept human supplementation study (NCT03838822). We observed that the supplementation resulted in increased fatty acid oxidation and de novo GSH synthesis [20]. However, despite these very promising results, the chronic effects of this multi-ingredient supplement including the four metabolic activators have not been yet evaluated, and, even more important, the underlying molecular mechanism associated with this multi-ingredient supplementation has not been fully elucidated in animal or human studies.

In the present study, we investigated the effect of the chronic administration of a CMA-based multi-ingredient supplement, including NR, LCT, NAC, and betaine (as a GSH precursor because it can be converted to glycine and serine, one of the most significant changed metabolites in subjects with high HS), in Golden Syrian hamsters with NAFLD induced by HFD. We used Golden Syrian hamsters because they are a better model for experimental studies on lipoprotein metabolism than other smaller-sized animals, e.g., rat or mouse [21,22,23], and NAFLD is often accompanied by dyslipidemia [5]. The CMA-based multi-ingredient supplement was given at two extrapolated human equivalent doses that can be considered acceptable and safe in a context of a multi-ingredient supplementation to tackle NAFLD. We found that the administration of CMA ameliorated HS at both doses when compared to the high-fat diet HFD counterparts. To explore the underlying molecular mechanisms involved in decreased HS, we generated liver transcriptomics data before and after administration of CMA and integrated these data using a liver genome-scale metabolic model (GEM). Finally, we systematically identified the phenotypical differences and presented the potential effects of CMA supplementation on the global metabolism based on systems analysis, showing that CMA would exert its hepatic effects activating mitochondria in the liver by modulating global lipid, amino acid, antioxidant and folate metabolism.

## 2. Materials and Methods

### 2.1. Animal Model

The Animal Ethics Committee of the Technological Unit of Nutrition and Health of Eurecat (Reus, Spain) and the Generalitat de Catalunya approved all procedures (DAAM 10026). The experimental protocol complied with the ARRIVE guidelines followed the ‘Principles of laboratory animal care’ and was carried out in accordance with the EU Directive 2010/63/EU for animal experiments. All animals were housed individually at 22 °C under a light/dark cycle of 12 h (lights on at 09:00 a.m.) and were given free access to food and water.

Thirty-nine 10-week-old male Golden Syrian hamsters (Janvier Labs, Saint Berthevin, France) weighting 110–120 g were used. After an adaptation period of 1 week, hamsters were randomly assigned into two experimental groups fed with a normal diet (NFD, *n* = 9, 11% calories from fat; Envigo, Barcelona, Spain) or an HFD (*n* = 30, 23% calories from fat and 1 g/kg cholesterol; Envigo, Barcelona, Spain) (Dataset S4) for 8 weeks. In a previous study using very similar diets, we demonstrated that this period was useful to induce fatty liver and hypercholesterolemia in hamsters [24]. Afterwards, the HFD group was further randomly distributed into three subgroups: placebo, CMA at Dose I (200 mg/kg LC, 200 mg/kg NAC, 200 mg/kg NR and 400 mg/kg betaine; HFD + MI_D1 group) and Dose II (400 mg/kg LC, 400 mg/kg NAC, 400 mg/kg NR and 800 mg/kg betaine; HFD + MI_D2 group) for the last 4 weeks. LCT contained 68.2% of LC and, therefore, to reach the desired LC doses, hamsters were supplemented with 293 mg/kg and 586 mg/kg of LCT for Dose I and Dose II, respectively. LCT (Cambridge Commodities, Ely, UK), NAC (Cambridge Commodities, Ely, UK) and NR (ChromaDex, Los Angeles, CA, USA) were diluted daily with low-fat condensed milk diluted 1:3 with water (vehicle) and orally given to the hamsters. Four days before the beginning of the treatments, the animals were trained to lick diluted low-fat condensed milk diluted 1:3 with water (0.2 mL) to ensure voluntary consumption. It was confirmed that each hamster fully ingested the daily dose of the corresponding treatment, which was given daily between 8:30–10:00 a.m. Betaine (Cambridge Commodities, Ely, UK) was included in opaque bottles containing the drinking water (4 g/L and 8 g/L for Dose I and Dose II, respectively) and renewed three times per week. Considering an average hamster’s weight of 125 g, the doses of LCT, NAC and NR used were equivalent to the daily consumption of 1918 mg and 3836 mg of these metabolic activators for a 60-kg human [25], for the Dose I and Dose II, respectively. For betaine, the extrapolated daily intake using the same formulas was 3836 mg and 7672 mg, respectively. These dosages were considered acceptable and safe in the context of a multi-ingredient supplementation to tackle NAFLD [19].

Body weight and food intake were recorded once per week, and food was renewed daily. At 12 weeks, all experimental animals were sacrificed under anesthesia (pentobarbital sodium, 60 mg/kg body weight) after 6 h of diurnal fasting. Blood was collected by cardiac puncture, and serum was obtained by centrifugation and stored at −20 °C until analysis. The liver, soleus and gastrocnemius muscles, and the mesenteric white adipose tissue depot (MWAT) were rapidly removed, weighed, frozen in liquid nitrogen and stored at −70 °C until analysis.

### 2.2. Histological Evaluation

Morphometric analyses of tissues and steatosis of liver histology were described as before [26]. Liver samples (*n* = 9–10 per group) were fixed in 4% diluted formaldehyde. After 24 h of fixation, successive dehydration (alcohol/ethanol 70%, 96% and 100%; plus xylol/dimethyl benzene) and paraffin infiltration-immersion at 52 °C (Citadel 2000, Thermo Scientific, Waltham, MA, USA) were performed. Training paraffin block (Histostar, Thermo Scientific) and subsequent successive 2 μ thickness sections (Microm HM 355S, Thermo Scientific) were prepared. Sections were deposited above slides (JP Selecta Paraffin Bath; Barcelona, Spain) and subjected to automated hematoxylin-eosin staining (Varistain Gemini. Shandom, Thermo Scientific).

Two independent researchers who were blinded to diet and treatment groups performed histological examination with a Leica DM750 microscope (Wetzlar, Germany), and the image was taken with Leica ICC50W camera (Software Leica Application Suite Version 3.3.0). The entire histological section (approximate area 2 cm^2^) of the liver was analyzed according to Kleiner score [27], estimating the percentage of the area covered by fat droplets and using a score from 0 and 3 (0, when steatosis was detected in <5% of the area; 1, when steatosis was detected in 5–33% of the area; 2, when steatosis was observed in 33–66% of the area; and 3, when steatosis was observed in more than 66% of the area).

### 2.3. Hepatic Lipid Extraction and Quantification

Lipid extraction and total lipid and triacylglycerol quantification were carried out using the methods described in the literature [28,29], with the modifications described in in-house literature [30]. Briefly, liver samples (80–100 mg) were mixed with 1 mL of hexane:isopropanol (3:2, *v*/*v*). The tubes with the samples were gassed with nitrogen before being closed to minimize lipid oxidation and then left overnight under orbital agitation at room temperature protected from light. The content of each tube was transferred into a new one, and 0.3 mL of Na_2_SO_4_ (0.47 M) was added. Tubes were mixed for 5 min, left for 15 min in orbital agitation, and centrifuged at 1000× *g* for 10 min at 4 °C. The upper phase containing lipids was dissolved in hexane and transferred to a clean, previously weighed glass tube. The hexane extract was then dried with nitrogen gas. Once the tube was dried, the percentage of lipids was determined gravimetrically. To evaluate triacylglycerol content, the lipid extracts (obtained as commented above) were dissolved in 1 mL of LPL buffer (28.75 mM Pipes; 57.41 mM MgCl_2_.6H_2_O; 0.569 mg/mL BSA-FFA free) with 0.1% SDS. Afterwards, samples were sonicated for 30 s, and tubes were left overnight in an orbital shaker at room temperature and protected from light. On the following day, the tubes were cold-sonicated with three pulses of 30 s each, and their triacylglycerol levels were measured immediately using an enzymatic colorimetric kit (QCA, Barcelona, Spain).

### 2.4. Body Composition Analyses

Body composition was analyzed by nuclear magnetic resonance (NMR). Lean and fat mass analyses were performed at the end of weeks 8 and 12 using an EchoMRI-700^®^ device (Echo Medical Systems, L.L.C., Houston, TX, USA). The measurements were performed in duplicate. Data were expressed in relative values as a percentage of body weight (%). Lean/fat mass ratio was also calculated.

### 2.5. Serum Analysis

Enzymatic colorimetric assays were used for the analysis of glucose, total cholesterol and triacylglicerols (QCA, Barcelona, Spain), HDL-cholesterol and LDL/VLDL-cholesterol (Bioassay systems, Hayward, CA, USA). Serum insulin levels were analyzed using a hamster insulin ELISA kit (MyBiosource, Bizkaia, Spain).

### 2.6. ^1^H NMR Analyses for NAD^+^, Sarcosine and Ascorbate Determination

For ^1^H NMR analysis, the metabolite aqueous extracts obtained in the liver were reconstituted in 700 μL of a solution containing trisilylpropionic acid (TSP) (0.74 mM) dissolved in D2O phosphate buffer (0.05 M). Samples were vortexed, homogenized for 5 min and centrifuged (15 min at 14,000× *g*). Finally, the redissolved extractions were transferred into 5 mm NMR glass tubes. ^1^H NMR measurements were performed following the procedure described by Vinaixa et al. (2010) [31].

### 2.7. Transcriptomics and Gene Set Enrichment Analysis

Total RNA was extracted from the liver samples using TriPure reagent (Roche Diagnostic, Sant Cugat del Valles, Barcelona, Spain) according to the manufacturer’s instructions, and used in the subsequent mRNA sample preparation for sequencing using NovaSeq 6000 S1 platform with the standard Illumina RNA-seq protocol. The RNA-seq was performed at National Genomics Infrastructure (NGI, Stockholm, Sweden) (project number P15763). Gene abundance (in both raw counts and transcripts per million) was quantified using the Kallisto pipeline [32] based on Golden Syrian hamster (*Mesocricetus auratus*) genome (version MesAur1.0.101). In order to validate the intra-group homogeneity, we first performed a principal component analysis (PCA). We filtered out one sample in control group based on the results of PCA analysis. Next, we used DESeq2 package in R [33] to identify differentially expressed genes (DEGs). We performed Benjamini–Hochberg false discovery rate (FDR) test to control for false positives. All the genes with an FDR derived q-value less than 0.1 (adjusted *p* value < 0.1) were identified as DEGs, suggesting that less than 10% of DEGs selected by this *p*-value could be false positives. Gene ontology and KEGG pathway analysis were performed separately on up-regulated and down-regulated genes using DAVID Bioinformatics Functional Annotation Tool [34]. Only ‘biological process’ categories enriched with a BH-corrected *p* ≤ 0.05 were considered for GO category analysis.

### 2.8. Metabolic Model Reconstruction and Reporter Metabolite Analysis

To provide a resource for automated and semi-automated reconstruction of liver-specific GEMs for Golden Syrian hamster, we constructed *iHamsterHepatocyte1818* based on the MMR databases [35], transcriptomics data, and Integrative Network Inference for Tissues (INIT) algorithm that has been used to reconstruct functional GEMs based on bulk transcriptomics data as well as 56 user-defined metabolic tasks, which are known to occur in cells/tissues [36] and should be performed by the resulting metabolic model. A reporter metabolite analysis was performed using RAVEN toolbox implemented in MATLAB 2020a based on DEGs obtained as described above, together with *iHamsterHepatocyte1818*.

### 2.9. Statistical Analysis

The continuous variables of biological assays were shown as mean ± SD. Grubbs’ test was used to detect outliers, which were discarded for subsequent analyses. The assumption of normality was determined using the Kolmogorov–Smirnov test, and the homoscedasticity among groups was assessed using Levene’s test. When one or both of these conditions were not met, data were transformed to a base-10 logarithm to obtain a normal distribution and/or similar variances before statistical testing. Differences in energy intake and biometric and serum parameters between the NFD-fed (control) hamsters and the HFD-fed hamsters after 8 weeks were detected using a non-parametric Mann–Whitney-Wilcoxon test because data were not normally distributed. At the end point (week 12), one-way ANOVA followed by Tukey’s post hoc test were used to evaluate differences in energy intake, hepatic lipid content, biometric parameters and serum parameters among control, HFD, HFD + MI_D1 and HFD + MI_D2 groups. Differences in steatosis scores were detected using the Chi-squared test. Principal component analysis (PCA) was performed on transcriptomics data sets to explore the quality of data and detect possible outliers. All results were considered statistically significant at *p* < 0.05. Statistical analyses were performed using Python 3.7. R (v4.0.3) was used for data visualization.

## 3. Results

### 3.1. CMA Attenuated HFD-Induced HS in a Golden Syrian Hamster NAFLD Model

**Figure 1:**
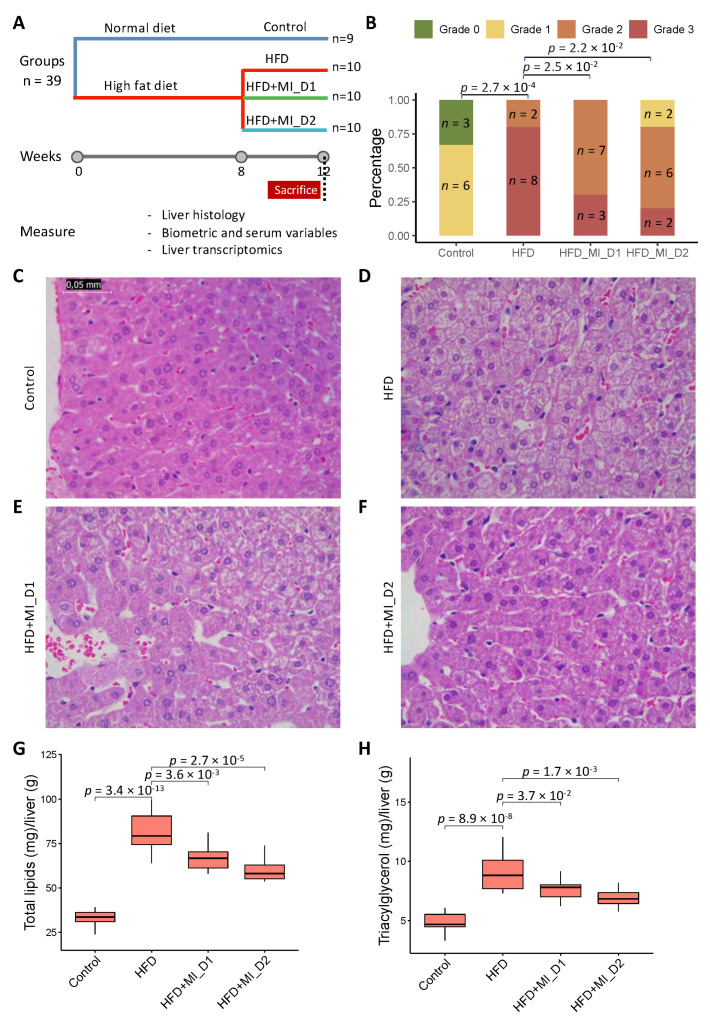
Effect of HFD and CMA treatment with two doses on liver histology of Golden Syrian hamsters. (**A**) Study design. (**B**) Steatosis score of histological changes in liver. (**C**–**F**) Histological analysis of steatosis in liver sections stained with Hematoxylin and Eosin (400 ×). (**G**) Relative hepatic total lipid content of hamsters in experimental groups. (**H**) Relative hepatic triacylglycerol level of hamsters in experimental groups. Control, normal diet; HFD, high-fat diet; HFD + MI_D1, high-fat diet supplemented with CMA at Dose I; HFD + MI_D2, high-fat diet supplemented with CMA at Dose II. Differences among groups in steatosis score were assessed using a Chi-squared test. Differences among groups in hepatic total lipid content and triacylglycerol level were derived from one-way ANOVA followed by Tukey’s post-hoc test. All results were considered statistically significant at *p*-value < 0.05.

We fed Golden Syrian hamsters with either an NFD or HFD for 8 weeks. Afterwards, we provided the animals either a placebo or a CMA including NR, LCT and NAC by oral gavage and betaine in their drinking water at intended human clinical doses (Dose I; HFD + MI_D1 group) and 2-fold (Dose II; HFD + MI_D2 group) for 4 weeks (Figure 1A). We assessed the changes in body weight, biometric and serum variables of each hamster group at week 8 (after 8 weeks of NFD or the HFD intervention) and week 12 (after 4 weeks of the treatment with the placebo or the CMA at two doses) and examined the phenotypic differences in all groups.

As expected [24], at week 8, HFD hamsters displayed a significant increase in circulating levels of total cholesterol when compared with their control counterparts (Table 1). No significant changes were found at this time point between groups either in cumulative energy intake or in biometric parameters (Table 1).

We observed that, at week 12, the hamsters fed with HFD had a significantly more-severe grade of HS (measured as steatosis score after histological analysis) compared to those from the NFD-fed group (*p* < 0.001), and this HFD-induced HS was significantly attenuated in CMA-treated hamsters with Doses I and II in comparison to their HFD counterparts (Chi-squared test; *p* = 0.025 and *p* = 0.022, respectively) (Figure 1B). Additionally, the histological analyses showed a significant increase in lipid deposition in the HFD group’s liver samples compared to those of the control group (Figure 1C,D). Specifically, the HFD groups developed microvesicular steatosis without apparent inflammation, sinusoidal dilatation or fibrosis (the latter visually determined) (Figure 1D), and CMA supplementation with Dose I and II reduced the HFD-induced lipid deposition in the liver (Figure 1E,F). In line with the histological examination, the biochemical analyses revealed that the HFD-fed hamsters displayed a significant increase in both lipid and triacylglycerol hepatic content than their control counterparts. The increases in these parameters were partially counteracted by the supplementation with CMA at both doses (Figure 1G,H).

We also evaluated the related phenotypes of the hamsters after supplementation (week 12) with CMA or the placebo (Table 2). At the end of the study, the HFD-fed hamsters, compared to the control group, showed significantly increased absolute and relative liver weights (adjusted-*p* = 0.013 and adjusted-*p* = 0.008, respectively) (Table 2) and relative mesenteric adipose tissue depots (MWAT%, mean increase 37.5%, *p* = 0.03) (Table 2). CMA treatment tended to induce a reduction in absolute liver weight, mainly in hamsters supplemented with Dose II (8.8% and 10.1% reduction with Dose I and II, respectively) compared to the HFD group, although the differences were not statistically significant (*p* > 0.05) (Table 2). The monitoring of biometric parameters revealed that there were no significant differences in body weight, fat mass, lean mass, and lean/fat ratio between hamsters fed the HFD and those fed the NFD. However, hamsters treated with CMA and Dose II showed significantly lower body weight gain (*p* = 0.0002) than the HFD group (Table 2).

At the end of the study, hamsters in the HFD group displayed significantly higher plasma levels of total cholesterol (CHOL; *p* = 0.0019) and low-density lipoprotein cholesterol (LDL-C; *p* = 0.0022) than their control counterparts at week 12 (Table 2). The CMA supplementation with the two doses did not ameliorate the increase observed in these parameters after the HFD challenge (Table 2).

### 3.2. Transcriptomics Alteration with HFD and CMA Treatment

**Figure 2:**
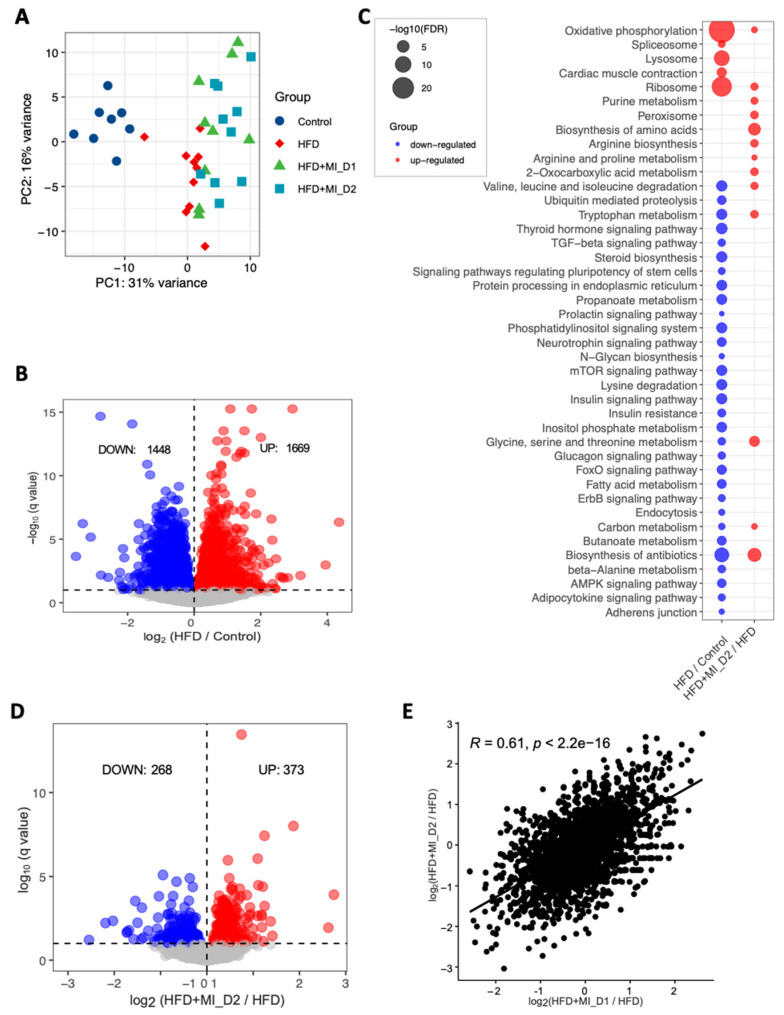
Effects of HFD and CMA treatment with two doses on liver transcriptomics of Golden Syrian hamsters. (**A**) PCA plot of 37 hamsters. (**B**) Up-and down-regulated genes in HFD group as compared to control group. (**C**) KEGG pathway analysis shows pathways that were significantly altered between HFD and control groups, HFD + MI_D2 and HFD groups, respectively. Pathways that were down-regulated or up-regulated are shown in blue and red, respectively. The size of the bubble is proportional to −log10 of the FDR for each KEGG pathway term. (**D**) Up-and down-regulated genes in HFD + MI_D2 group as compared to HFD group. (**E**) The log 2-fold changes in differential expressed genes between two groups compared to HFD group were significantly positively correlated.

To reveal the underlying molecular changes associated with the decreased HS after CMA treatment, we performed global transcriptomics analysis using RNA sequencing (RNA-seq) on liver tissue of each group. Globally, PCA revealed patterns of gene expression changes with HFD and CMA treatments (Figure 2A). At a 10% false discovery rate (FDR), we found 3317 differentially expressed genes (DEGs) between the HFD and control groups, 1669 of which were significantly up-regulated and 1648 of which were down-regulated (Figure 2B, Dataset S1). We performed KEGG pathway enrichment analysis on up-regulated and down-regulated DEGs separately in each group. Our results indicated that up-regulated DEGs were enriched in pathways including oxidative phosphorylation, ribosome, metabolic pathway, lysosome, cardiac muscle contraction, and spliceosome in HFD group vs. control group (Figure 2C, Dataset S2). We found that fatty acid metabolism, catabolism of amino acids including branched-chain amino acids (BCAAs) and lysine, metabolism of amino acids (tryptophan, beta-alanine, glycine, serine, and threonine), as well as signaling pathways in the regulation of insulin resistance and fatty acid oxidation (e.g., AMPK-signaling pathway) were significantly enriched with the down-regulated DEGs in HFD group vs. control group (Figure 2C, Dataset S2).

We also performed gene ontology (GO) enrichment analysis for the DEGs. Compared to the control group, we found that up-regulated genes in the HFD group were most significantly enriched in nucleoside monophosphate metabolic process, ATP synthesis coupled electron transport, respiratory electron transport chain and purine nucleoside metabolic process. We also found that down-regulated genes were most significantly enriched in cellular protein modification process, protein modification process and cellular protein metabolic process in HFD vs. control group (Dataset S2).

Next, we compared the gene expression changes between HFD and CMA-treated groups (fed with HFD + MI_D1 and HFD + MI_D2). In total, 80 (56 up-regulated and 24 down-regulated) and 641 DEGs (268 down-regulated and 373 up-regulated) (Figure 2D, Dataset S1) were identified in the Dose I and II groups, respectively. We found that the log 2-fold changes in DEGs between two groups compared to HFD group were significantly positively correlated (*r* = 0.61, *p* < 2.2 × 10^−^^16^) (Figure 2E). This suggested that the supplementation with different dosages has a very similar effect on hamster liver. Thus, we focused on the Dose II group in the following analyses, as it demonstrated more distinct and significant changes associated with the supplementation of CMA. The KEGG enrichment analysis showed that 14 pathways were up-regulated after 4-week supplementation of CMA at Dose II (Figure 2C, Dataset S2). Of these, the most up-regulated pathways involved biosynthesis and metabolism of amino acids, including glycine, serine, threonine, tryptophan, alanine, aspartate glutamate, arginine and proline, as well as BCAAs (Figure 2C, Dataset S2). GO enrichment analysis of up-regulated DEGs showed that the oxoacid metabolic process, carboxylic acid metabolic process, and alpha-amino acid metabolic process were the most significantly altered biological processes (Dataset S2) in the Dose II group vs. the HFD group.

### 3.3. Reporter Metabolites through the Global Analysis of Transcriptomics Data

**Figure 3:**
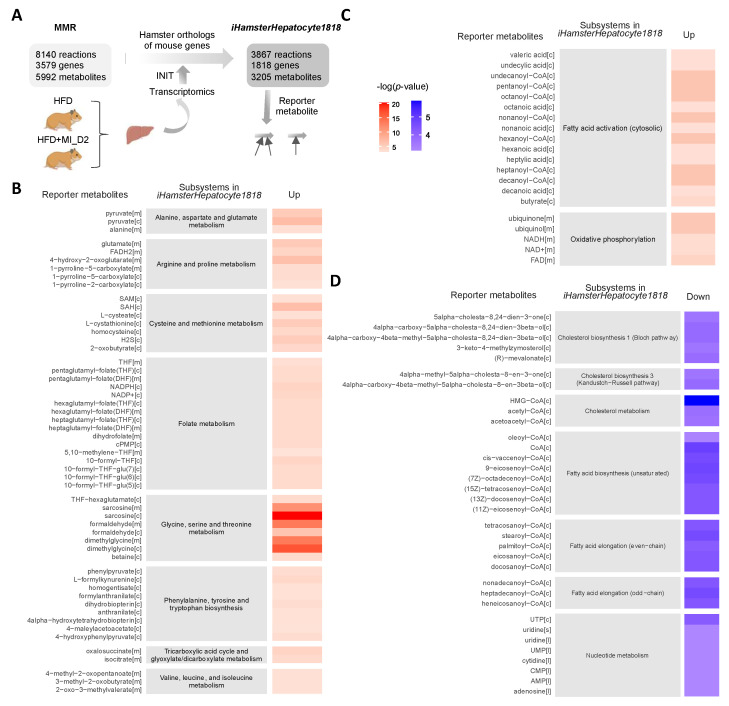
Reporter metabolites in CMA-treated group with Dose II. Metabolic differences between the hamsters treated with or without CMA at Dose II (HFD and HFD + MI_D2) were investigated through a comparative analysis of the gene expression profiles (RNA-seq) of the liver and *iHamsterHepatocyte1818*. (**A**) Liver-specific genome-scale metabolic model for Golden Syrian hamster (*iHamsterHepatocyte1818*) was created using the Golden Syrian hamster orthologs of mouse genes based on the mouse metabolic reaction (MMR); (**B**) reporter metabolites associated with amino acid metabolism and tricarboxylic acid (TCA) cycle; (**C**) fatty acid activation and oxidation phosphorylation; (**D**) biosynthesis and metabolism of cholesterol, biosynthesis and elongation of fatty acid, and nucleotide metabolism. *p* values for each reporter metabolite were calculated for up-and down-regulated genes. Abbreviations: CMA, combined metabolic activators; INIT, integrative network inference for tissue.

To evaluate the detailed metabolic differences in hamsters with or without CMA treatment upon HFD feeding, we constructed a liver-specific genomic-scale metabolic model for Golden Syrian hamster based on the Golden Syrian hamster orthologs of mouse genes in mouse metabolic reaction (MMR) [35], and transcriptomics data by using the Integrative Network Inference for Tissues (INIT) algorithm [37] (Figure 3A). The constructed metabolic model, namely iHamsterHepatocyte1818, included 3867 metabolic reactions, 1818 genes, and 3205 metabolites.

Next, we performed reporter metabolite analysis to identify the key metabolic hubs in response to the supplementation of CMA with Dose II. We identified reporter metabolites using the differentially expressed genes of Dose II vs. HFD group and the network topology provided by iHamsterHepatocyte1818. Reporter metabolite analyses were used as a statistical test to identify metabolites in the network for which a significant change occurred between the compared conditions. A total of 256 reporter metabolites (reporter features, *p*-value < 0.05) for hamsters treated with CMA with Dose 2 were identified (Dataset S3). Of these, 143 and 113 reporter metabolites were associated with up-regulated and down-regulated genes in the CMA-treated group, respectively. The association of the reporter metabolites with up-and down-regulated genes and their metabolic subsystems classified in iHamsterHepatocyte1818 is presented (Figure 3B–D). As shown in the figure, CMA treatment modulated several subsystems that are known to be associated with NAFLD, including amino acid metabolism (BCAAs, cysteine, methionine, glycine, serine, threonine, arginine, proline, alanine, aspartate, and glutamate), folate metabolism, tricarboxylic acid cycle (TCA) (Figure 3B), fatty acid activation and oxidative phosphorylation (Figure 3C). In addition, CMA supplementation regulated the subsystems associated with biosynthesis and metabolism of cholesterol, biosynthesis and elongation of fatty acid, as well as nucleotide metabolism (Figure 3D).

**Figure 4:**
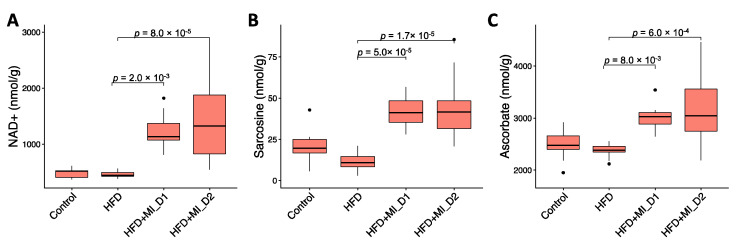
Hepatic concentration (nmol/g) of key metabolites modulated by CMA. (**A**) NAD^+^ hepatic levels; (**B**) sarcosine hepatic levels; (**C**) ascorbate hepatic levels. Statistical differences were obtained by one-way ANOVA followed by Tukey’s post-hoc test.

To validate our metabolic modeling approach, we measured, using NMR, the hepatic levels of two key metabolites associated with CMA supplementation, namely NAD^+^ and sarcosine. We observed that both NAD^+^ and sarcosine hepatic levels were significantly increased with CMA Dose I and II supplementation compared to their HFD counterparts (Figure 4A,B), as predicted in our earlier studies. Furthermore, our NMR analysis also revealed a significant increase in the hepatic concentrations of the potent antioxidant ascorbate in both HFD + MI_D1 and HFD + MI_D2 groups when compared to their HFD counterparts (Figure 4C).

### 3.4. CMA Boosted Hepatic Metabolism to Attenuate HS

**Figure 5:**
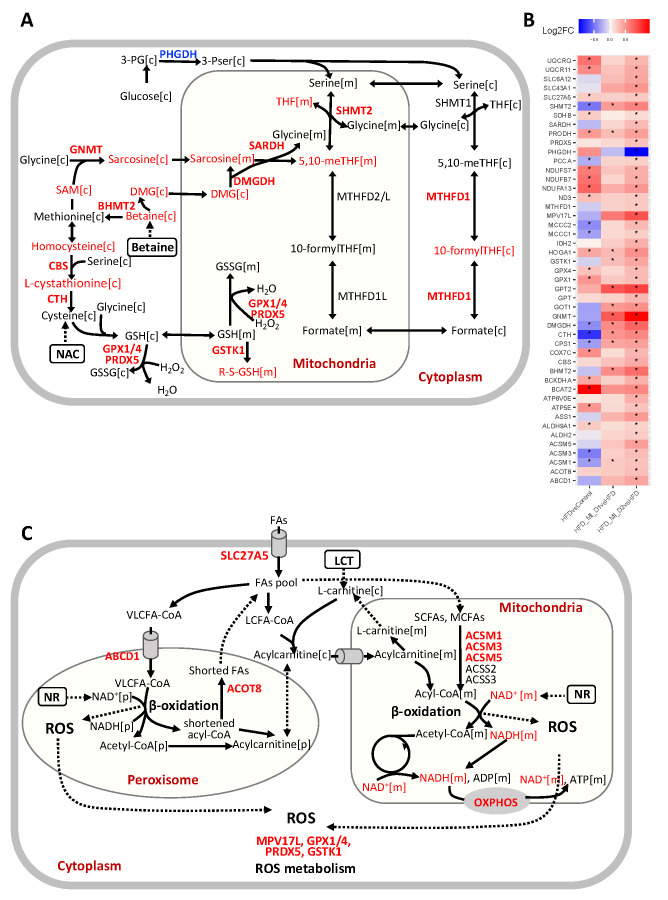
Significant increases (red) and decreases (blue) in genes and associated reporter metabolites in liver from hamsters in HFD + MI_D2 as compared to those in HFD group. (**A**) Reactions involved in one-carbon metabolism and transsulfuration pathway. (**B**) A summary of significantly expressed genes involved in one-carbon metabolism, transsulfuration pathway, fatty acid metabolism, oxidative phosphorylation (OXPHOS), BCAA degradation, TCA cycle and urea cycle. * Demonstrates the significance (adjusted *p*-value < 0.1). (**C**) Reactions involved in fatty acid metabolism in both mitochondria and peroxisome. Abbreviations: DMG, dimethylglycine; THF, tetrahydrofolate; SCFAs, short-chain fatty acids; MCFAs, medium-chain fatty acids; LCT, L-carnitine tartrate; GSH, glutathione; GSSG, glutathione disulfide; 3-PG, 3-phosphoglycerate; 3PSer, 3-phosphoserine; SAM, S-Adenosyl-l-methionine.

Based on the integrative systems analysis, we found the CMA supplementation enhanced several NAFLD-associated key metabolic pathways in the liver. We found that CMA supplementation, or more specifically, betaine supplementation, increased hepatic expression of genes involved in folate metabolism, including *SHMT2*, *MTHFD1*, *DMGDH*, *SARDH* and *GNMT* (Figure 5A,B). This implicated an enhanced folate cycle in the liver. Additionally, we observed a significant increase in the hepatic expression of *CBS* and *CTH* involved in transsulfuration pathway, where the supplemented NAC might play an important role (Figure 5A,B). This may contribute to GSH generation by synthesizing cysteine and may play a crucial role in maintaining cellular redox homeostasis [38]. We also observed increased hepatic expression of key genes involved in mediating GSH metabolism, including *GPX1*, *GPX4*, *GSTK1*, and *PRDX5* (Figure 5A,B). Based on reporter metabolite analysis, we also identified betaine, dimethylglycine, sarcosine, 5,10−methylene−THF, 10−formyl−THF, THF, homocysteine, L-cystathionine and S-(formylmethyl) glutathione, which are associated with up-regulated genes in folate metabolism, the transsulfuration pathway and glutathione metabolism (Figure 3B, Figure 5A,B). Interestingly, the increase in hepatic levels of sarcosine (also known as N-methylglycine) found in hamsters supplemented with CMA at both doses was in agreement with our predictions of enhancement of the subsystems of glycine, serine and threonine metabolism, including the metabolite sarcosine, in response to the administration of this multi-ingredient (Figure 3B, Figure 4B). Considering that one of the significant components of the CMA is betaine, which is a precursor for these metabolic pathways (Figure 5A), these results suggested that the supplementation promoted glutathione biosynthesis through the folate cycle and transsulfuration pathway.

Peroxisome and mitochondria cooperatively perform diverse metabolic processes, including fatty acid β-oxidation and cellular ROS homeostasis [39]. Short-and medium-chain fatty acids (SCFAs, MCFAs) are oxidized in the mitochondria, whereas long-chain fatty acids (LCFAs) are oxidized in both mitochondria and peroxisome. Very-long-chain fatty acids (VLFAs) are preferentially oxidized in the peroxisomes [40]. We found that the hepatic expression of genes associated with fatty acid oxidation in peroxisome and mitochondria (*SLC27A5*, *ABCD1*, *ACOT8*, *ACSM1*, *ACSM3*, and *ACSM5*) were elevated in the liver of CMA-treated hamsters, which could be related to the supplementation of LCT and NR (Figure 5B,C). Moreover, we observed that the genes involved in ROS metabolism, including *MPV17L*, *GSTK1*, *PRDX5*, *GPX1*, and *GPX4* (Figure 5B,C), showed a significant increase, which could be a result of the supplemented NR and elevated glutathione metabolism. *SLC27A5*, which facilitates the uptake of LCFAs, has been considered as long-chain and very-long-chain acyl-CoA (VLCFA-CoA) synthetases [41,42]. Interestingly, the hepatic expression of genes involved in de novo synthesis of NAD^+^, an important coenzyme for redox reactions, including *TDO2*, *AFMID*, and *KYNU*, was significantly increased after CMA supplementation. NAD^+^ and NADH were also identified as reporter metabolites associated with up-regulated genes in fatty acid oxidation, oxidative phosphorylation and TCA cycle (Figure 5B,C). Notably, in agreement with the aforementioned findings, we found that the NAD^+^ level was significantly increased in the liver with CMA Dose I and II supplementation vs. HFD (Figure 4A). In addition, we found that the hepatic levels of ascorbate, a well-known potent antioxidant, were significantly increased with CMA Dose I and II supplementation compared to that in HFD counterparts. Ascorbate reduces the content of reactive oxygen species in hepatocyte mitochondria scavenging superoxide, hydroxyl and peroxyl radicals, and inactivating hypochlorite and singlet oxygen [43]. Ascorbate also improves the reduced glutathione (GSH) status by recycling oxidized glutathione and can improve mitochondrial function by improving the thiol status [43,44]. Different types of scientific evidence obtained in animal studies as well as through epidemiological surveys have demonstrated that ascorbate deficiency is correlated with increasing risks for NAFLD [45,46,47,48]. Furthermore, ascorbate supplementation ameliorated HS induced by a high palm oil diet in guinea pigs [49] and improved hepatic mitochondria function in arsenic-treated rats [50].

**Figure 6:**
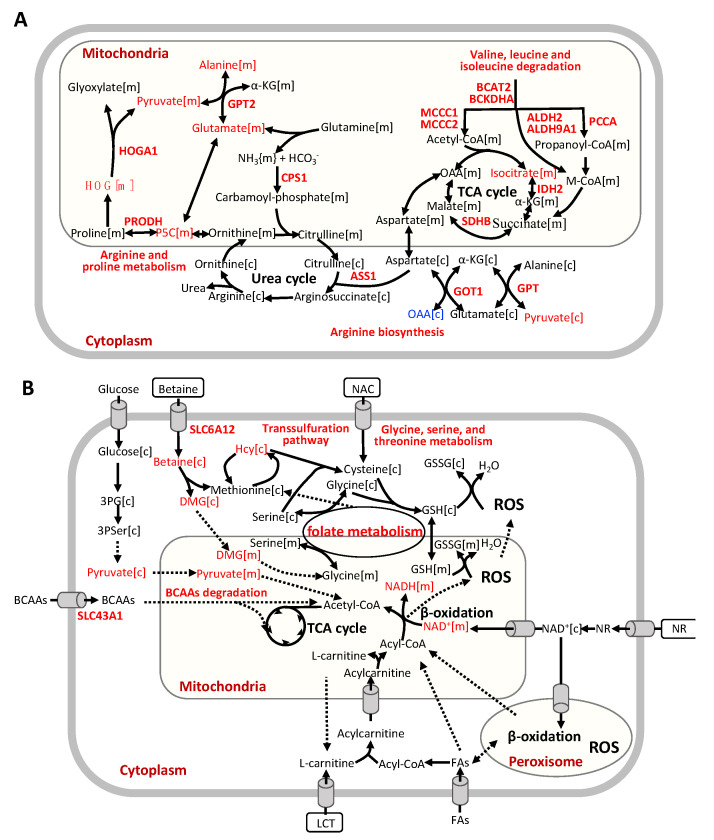
(**A**) Reactions involved in BCAA degradation, tricarboxylic acid cycle, and urea cycle. (**B**) Global effect of CMA supplementation. Abbreviations: BCAAs, branched-chain amino acids; OAA, Oxalacetic acid; α-KG, alpha-ketoglutarate; HOG, 4−hydroxy−2−oxoglutarate; M-CoA, methylmalonyl-CoA; P5C, 1−pyrroline−5−carboxylate; Hcy, homocysteine; GSH, glutathione; GSSG, glutathione disulfide; 3-PG, 3-phosphoglycerate; 3PSer, 3-phosphoserine; DMG, dimethylglycine; NR, nicotinamide riboside; NAC, N-acetyl-l-cysteine; FAs, fatty acids; LCT, L-carnitine tartrate.

Additionally, we found that CMA supplementation increased the hepatic expression of genes involved in BCAA catabolism, including *BCAT2*, *BCKDHA*, *MCCC1*, *MCCC2*, *ALDH2*, *ALDH9A1* and *PCCA* (Figure 5B, Figure 6A). We also observed significantly enhanced hepatic expression of *IDH2* and *SDHB* in TCA cycle. Increased TCA cycle and BCAA catabolism could indicate that the fatty acid oxidation and cellular respiration are enhanced as suggested by a previous study [51]. Moreover, we observed that the hepatic expression of genes involved in arginine biosynthesis (*GPT*, *GOT1*, *GPT2*, *CPS1* and *ASS1*) and genes involved in arginine and proline metabolism was significantly increased with the CMA supplementation. It has been reported that the down-regulation of *CPS1*, the flux-generating urea cycle feeder enzyme, correlated with the loss of functional capacity for ureagenesis in patients with NASH [52,53].

## 4. Discussion

In this study, we evaluated the global changes induced by the CMA treatment (Figure 6B). We demonstrated that HFD-fed hamsters that received the CMA at both doses displayed an amelioration of HS compared to their HFD-fed control counterparts. Interestingly, our transcriptomics analysis revealed that after CMA treatment there was a significant increase in the hepatic expression of genes in one-carbon metabolism and transsulfuration pathway, which are closely associated with the de novo GSH synthesis, which, in turn, is altered in patients with HS [9]. Moreover, we reported that CMA supplementation increased the expression of genes involved in BCAA catabolism and the urea cycle in the liver. Furthermore, we observed, in hamsters supplemented with CMA at both doses, elevated NAD+ hepatic levels, the lack of which has a critical role in the development of NAFLD [9,54], as well as increased hepatic concentrations of the one-carbon metabolite sarcosine and free radical scavenger ascorbate. Altogether, our findings strongly suggest CMA supplementation as a very promising strategy to ameliorate fatty liver.

Our findings agreed well with, and also extended, the predicted results from our recent work [20]. Our previous studies revealed that altered GSH and NAD+ metabolism is a prevailing feature of NAFLD [9]. Amino acids, in particular serine, glycine, methionine, and several metabolites involved in one-carbon metabolism, play crucial roles in NAFLD progression [16]. Mice with HFD-induced NAFLD also showed dysregulated one-carbon metabolism in liver [55,56]. Dysregulated BCAA metabolism was significantly associated with the progression of NAFLD [57] and increased plasma levels of BCAAs were reported in NAFLD metabolomics studies [58]. Based on our previous study, we predicted that catabolism of BCAAs was significantly increased after CMA supplementation based on integration of GEMs and metabolomics data [20]. In patients with NAFLD, it has been reported that increased BCAA levels are likely linked to increased insulin resistance [59,60], and worsening insulin resistance in the setting of NAFLD is associated with the progressive inability of insulin to suppress plasma BCAAs, which in turn results in their elevated plasma levels [61,62]. In addition, BCAAs and their metabolites are involved in the optimal up-regulation of TCA cycle, since dysregulated BCAA metabolism in the setting of insulin resistance could impair the physiological induction of mitochondrial TCA cycle, leading to mitochondrial dysfunction in the progression of NAFLD [61]. In the current study, based on the integration of GEMs and transcriptomics data, we predicted that the catabolism of BCAAs was significantly increased after CMA supplementation, showing also increased hepatic mRNA levels of *BCAT2*, *BCKDHA*, *MCCC1*, *MCCC2*, *ALDH2*, *ALDH9A1* and *PCCA* in the HFD + MI_D2 hamsters. This is in line with our findings observed in a previous study in which the acute effect of a CMA including NAC, serine, NR and LCAT was evaluated in both rats and humans [20], reinforcing the idea that CMA would tackle NAFLD through the modulation of this metabolic pathway.

Serine hydroxymethyltransferase (*SHMT*) catalyzes the interconversion of glycine and serine, which are precursors for the generation of GSH, an important antioxidant for maintaining the redox balance in fatty acid β-oxidation [9,63]. Glycine N-methyltransferase (*GNMT*), the main gene involved in liver S-adenosylmethionine (SAM) catabolism, is down-regulated in the liver of HFD hamsters [64], as well as patients with cirrhosis and HCC [65]. In other studies, deletion of *GNMT* in mice led to excess increase in hepatic SAM, which is associated with fatty acid metabolism, oxidative stress and HS [66,67]. The down-regulation of *DMGDH*, which is a mitochondrial dimethylglycine dehydrogenase, was associated with insulin resistance in a previous study [68]. The hepatic *CBS/CTH* system was even positioned as a potential therapeutic target in NAFLD due to the deficiencies of *CBS* and *CTH* in rodent studies [69,70]. Taking into account the aforementioned findings [71], in the present study, the increased expression of key genes involved in folate metabolism (*SHMT2*, *MTHFD1*, *DMGDH*, *SARDH*, and *GNMT*) and in the transsulfuration pathway (*CBS* and *CTH*) found in the livers of CMA-supplemented hamsters strongly suggests that CMA ameliorated NAFLD promoting glutathione biosynthesis through the activation of these two metabolic pathways.

*IDH2* is considered a unique enzyme in the regulation of mitochondrial ROS [72]. *IDH2* knockout accelerates oxidative stress, lipid accumulation and HS in HFD-challenged mice, which were restored by promoting *IDH2* expression [73]. In the present study, we found that *IDH2* was up-regulated in the livers of hamsters after CMA supplementation vs HFD. Relevantly, the ROS metabolism-related genes *MPV17L* and *GSTK1* displayed the same pattern of expression than those observed for *IDH2*. *MPV17L*, a transmembrane protein, has been implicated in the regulation of peroxisomal ROS metabolism [74]. *MPV17L* also prevents mitochondrial dysfunction and apoptosis through its antioxidant and antiapoptotic properties in vivo and in vitro [75]. *GSTK1* is a highly conserved enzyme potentially involved in redox reaction and has a crucial role in protection against oxidative stress [76]. In addition, the expression of *GPX1* and *GPX4* genes, which code for the glutathione peroxidase (GPx) family of enzymes, was also up-regulated in the hamsters that received the CMA treatment. GPx catalyzes the reduction of H_2_O_2_ and lipid peroxides through the conversion of GSH to GSSG and thereby plays a crucial role in the protection of cells against oxidative damage. Patients with NASH had significantly lower levels of GPx activity [77]. Overall, our results strongly suggest that CMA would protect against NAFLD-associated oxidative stress, at least in part, through the up-regulation of the expression of these key genes involved in ROS metabolism. In addition, the increased hepatic concentrations of ascorbate found in HFD + MI_D1 and HFD + MI_D2 hamsters would provide extra evidence for the protective role of CMA against the oxidative stress that occurs in NAFLD. Nevertheless, up-regulation of gene expression is not always translated into increased protein activity or into changes in the concentration of functional metabolites. Therefore, further research focused on the analyses of hepatic GSH and ROS levels would be of great value to confirm that the modulation of the expression of both GSH-and ROS-related genes is translated into increased concentrations of GSH and decreased levels of ROS in the livers of CMA-supplemented hamsters.

The increased hepatic expression of genes associated with fatty acid oxidation in peroxisome and mitochondria (*SLC27A5*, *ABCD1*, *ACOT8*, *ACSM1*, *ACSM3* and *ACSM5*) found in response to the supplementation with CMA suggests that this multi-ingredient treatment would tackle NAFLD by boosting this catabolic pathway in both organelles. Different previous findings contribute to reinforce our hypothesis. Thus, a recent study showed that knockout of *SLC27A5* leads to disrupted lipid metabolism and redox balance in HCC cells both in vitro and in vivo [78]. In patients with severe steatohepatitis and cirrhotic liver, the hepatic expression levels of *SLC27A5* were significantly decreased and inversely correlated with histological progression [79]. *ABCD1* mediates the uptake of the VLCFAs across the peroxisome membrane, and its loss of function results in defective β-oxidation of VLCFA and increased cellular oxidative stress [80,81]. However, the mRNA levels of the gene encoding for carnitine palmitoyl transferase alpha (CPT1α), which plays a key role in the supply of fatty acids into the mitochondrial matrix, did not increase in the CMA-supplemented hamsters when compared to their HFD counterparts, which would not fully support our findings. One limitation of the gene expression data is the fact that they do not always match protein levels. Therefore, further studies of protein expression performed in liver would be useful to support our hypothesis. Furthermore, different studies have shown that the use of activators of AMPK (via phosphorylation) could be a useful strategy to improve NAFLD, boosting fatty acid oxidation by enhancing CPT1α flux [82]. Therefore, additional studies focused on the AMPK-signaling pathway in the liver could also contribute to clarifying if CMA can activate β-oxidation through the phosphorylation of AMPK. Nevertheless, the fact that our KEEG pathway analyses revealed a down-regulation of the AMPK-signaling pathway in the HFD animals when compared to the control group and that this pathway was not significantly up-regulated in the HFD + MI_D2 group vs. HFD hamsters would not be in agreement with this idea.

Epidemiologic data indicate that HS is associated with insulin resistance, and HS provokes an impairment of insulin action in skeletal muscle [83]. In addition, in mice challenged with an HFD, NAFLD preceded muscle lipid accumulation and whole-body hyperinsulinemia and hyperglycemia [83]. Furthermore, a recent study carried out with middle-aged Japanese subjects showed that the simultaneous presence of low skeletal muscle index (SMI) and density (SMD) was positively associated with a higher prevalence of NAFLD [84], highlighting the importance of muscle in NAFLD pathogenesis. In our study, CMA supplementation did not decrease the circulating levels of insulin and glucose and did not increase lean mass or the lean/fat mass ratio in HFD + MI_D1 and HFD + MI_D2 hamsters. These results would suggest, at first glance, that CMA would not ameliorate NAFLD by enhancing muscle accretion or muscle metabolism and that this multi-ingredient treatment would exert its beneficial effects mainly targeting the liver. However, further studies focused on muscle function would be needed to fully support this hypothesis.

Several studies have shown that the degree of weight loss is strongly associated with the reduction of NAFLD [85]. In this sense, a consensus document about the clinical practice guideline for the management of NAFLD stated that a weight loss of at least 7% by improving lifestyle or undergoing bariatric surgery over one year, improves NAFLD and its related complications [85]. Specifically, the authors concluded that a weight reduction between 7–10% in patients with few risk factors significantly reduces steatosis, inflammation and ballooning, also improving metabolic alterations, and that a weight loss of 10% or more could highly counteract the histological lesions occurring with NAFLD and improve its comorbidities [85]. Relevantly, in our study, we observed an 8.1% reduction of body weight and a significantly lower body weight gain in the animals supplemented with CMA at Dose II when compared to their HFD counterparts, which strongly suggest a strong potential of our multi-ingredient treatment to tackle NAFLD also through weight loss. This is also in agreement with our hypothesis to use multi-ingredient-based supplements that act against complementary targets, such as CMA, as potential strategies to effectively tackle this multifactorial disease, since different studies have claimed that better outcomes against NAFLD are obtained when treatments are performed under a multifaceted approach [7]. Three mechanisms responsible for the body weight loss produced by the supplementation of bioactive compounds are the inhibition of food intake, the enhancement of energy expenditure (EE), and the regulation of lipid metabolism [86]. Since we did not observe significant changes in energy intake in response to CMA treatment, it is plausible to hypothesize that the supplementation with CMA could decrease body weight gain through an enhancement of EE. This could be related to improved mitochondrial function and to the activation of thermogenesis in brown adipose tissue, which is, in rodents, the major contributor of energy dissipation through the production of heat [87]. Furthermore, an increased locomotor activity could also contribute to an increased EE. However, in the present study, we did not collect brown adipose tissue or carry out locomotor activity measurements, which would have been of interest to shed more light on this issue. In addition, the activation of β-oxidation and/or the inhibition of lipogenesis and fatty acid synthesis in different tissues, such as liver, muscle and adipose tissue, could also account for the body weight lowering effects observed in the hamsters supplemented with CMA. In this sense, as explained before, our hepatic transcriptomic analyses revealed and up-regulation of fatty acid oxidation-related genes in both mitochondria and peroxisome, which would be in agreement with our hypothesis. However, no significant changes in lipogenic genes such as diacylglycerol acyltransferase 1 and 2 (*DGAT1* and *DGAT2*), glycerol-3-phosphate acyltransferase (*GPAT*) or the master regulator of lipogenesis Peroxisome proliferator activated receptor gamma (*PPARg*) were found in this tissue after CMA treatment, which would not support that CMA exerts its body weight lowering effects through an inhibition of hepatic lipogenesis. Additional studies focused on gene and protein expression performed in liver, white and brown adipose tissue, and skeletal muscle would be useful to shed more light on this issue.

This study has strengths and limitations. The major strength of this study is that, based on an integrative analysis of transcriptomics and GEMs in the liver, we unraveled the mechanisms by which CMA exerts its beneficial effects against NAFLD, showing that CMA ameliorated HS by activating mitochondria in the hepatic tissue by modulating the global lipid, amino acids, antioxidant and folate metabolisms. Furthermore, we also demonstrated, using an NMR metabolomics approach, that CMA supplementation at both doses increased the hepatic levels of three key metabolites involved in NAFLD pathogenesis, namely NAD^+^, the one-carbon metabolite sarcosine and the potent antioxidant ascorbate. In addition, the effects of CMA were evaluated at two extrapolated human equivalent doses that could be considered acceptable and safe to tackle HS in order to increase the likelihood of a subsequent successful animal-to-human translation. However, the hepatic levels of GSH and ROS were not analyzed, which would be very useful to fully validate the transcriptomics-based hypothesis pointing towards a modulation of ROS and GSH-related pathways exerted by CMA. Furthermore, the analyses of key enzymes involved in hepatic β-oxidation, such as CPT1α and phosphorylated AMPK, would be needed to support that CMA treatment enhanced fat oxidation. In addition, further studies focused on other tissues involved in the etiology and pathogenesis of NAFLD, such as skeletal muscle and white adipose tissue, would be of interest to fully understand CMA effects. Notably, CMA have been tested in numbers of human clinical phase 2 and phase 3 studies [88,89,90]. We have shown that CMA activates mitochondrial metabolism and decrease liver fat in humans as it has been shown in this animal study.

## 5. Conclusions

In conclusion, the administration for 4 weeks with CMA supplementation ameliorated HS in HFD-fed Golden Syrian hamsters. To shed light on the metabolisms by which CMA exerted its effects, we performed liver transcriptomics analysis and analyzed the data using GEMs. We observed that CMA supplementation significantly attenuated HFD-induced HS by modulating NAD^+^ metabolism, increasing the hepatic concentrations of the free radical scavenger ascorbate and modulating the mRNA levels of key genes involved in GSH and ROS-related pathways, fatty acid oxidation, BCAA catabolism and urea cycle, eventually activating mitochondria. Altogether, our results demonstrated that CMA act against complementary targets that are of paramount importance to effectively manage NAFLD. Our findings provide extra evidence for the beneficial effects of a treatment based on these metabolic activators against NAFLD. Further randomized controlled clinical trials in humans that focus on the effectiveness of CMA against NAFLD will be of great interest to shed more light on this issue.

## Figures and Tables

**Table 1:** Energy intake, body composition and serum parameters of hamsters fed with a NFD (control group) or HFD (HFD group) at the 8th week of the study.

	Control (*n* = 9)	HFD (*n* = 30)
Cumulative Food Intake (kcal)	205.45 ± 14.08	194.73 ± 15.73
**Biometric variables**		
Body weight (g)	123.05 ± 5.14	121.16 ± 7.16
Fat mass (%)	11.94 ± 3.20	14.10 ± 2.42
Lean mass (%)	84.89 ± 3.26	84.12 ± 2.34
Lean/fat ratio	7.83 ± 3.22	6.19 ± 1.41
**Serum parameters**		
CHOL (mM)	3.90 ± 0.61	6.16 ± 0.91^$^

Data are shown as the mean ± SD. Different superscript letters indicate statistically significant differences (*p* < 0.05, Mann–Whitney–Wilcoxon test).

**Table 2:** Effects of HFD and multi-ingredient treatment at two doses (HFD + MI_D1 and HFD + MI_D2) on food intake and biometric and serum variables at the end of the study (week 12).

	Control (*n* = 9)	HFD (*n* = 10)	HFD + MI_D1 (*n* = 10)	HFD + MI_D2 (*n* =10)
Cumulative food intake (kcal) *	126.8 ± 14.4	111.1 ± 13.2	104.2 ± 15.9	95.2 ± 10.7
**Biometric variables**				
Body weight (g)	122.20 ± 5.62	126.57 ± 8.19	120.08 ± 10.32	116.28 ± 9.86
Body weight gain (g) *	−0.85 ± 4.17	5.50 ± 6.61	0.27 ± 5.26	−6.27± 5.38 ^b^
Liver weight (g) *	4.23 ± 0.30	5.10 ± 0.68 ^a^	4.69 ± 0.60	4.62 ± 0.61
Liver weight (%) *	3.49 ± 0.25	4.02 ± 0.30 ^a^	3.92 ± 0.43	3.98 ± 0.29
MWAT (%) *	0.88 ± 0.24	1.21 ± 0.24 ^a^	1.16 ± 0.29	1.14 ± 0.19
MUS	0.27 ± 0.03	0.25 ± 0.03	0.24 ± 0.03	0.24 ± 0.03
Fat mass (%)	11.28 ± 2.59	13.14 ± 2.78	14.84 ± 4.18	13.10 ± 2.87
Lean mass (%)	85.42 ± 2.39	85.74 ± 3.03	83.95 ± 4.04	85.22 ± 3.09
Lean/fat mass ratio	8.00 ± 2.13	6.84 ± 1.67	6.20 ± 2.19	6.83 ± 1.70
**Serum variables**				
Glucose (mM)	7.29 ± 1.50	9.42 ± 2.01	8.93 ± 1.42	9.27 ± 2.01
Insulin (mIU/L)	16.69 ± 2.92	16.26 ± 3.61	16.17 ± 1.34	16.78 ± 3.43
CHOL (mM) *	2.87 ± 0.21	4.03 ± 0.65 ^a^	3.77 ± 0.60	4.44 ± 0.85
HDL-C (mM) *	2.57 ± 0.36	3.15 ± 0.50	2.87 ± 0.52	3.32 ± 0.60
LDL-C (mM) *	0.90 ± 0.20	1.38 ± 0.32 ^a^	1.22 ± 0.24	1.34 ± 0.28
TG (mM)	1.07 ± 0.30	1.15 ± 0.53	0.96 ± 0.31	0.82 ± 0.32

Data are shown as the mean ± SD. * indicates significant difference (*p* < 0.05) among groups detected by one-way ANOVA. a and b indicate difference in HFD vs. control groups and HFD + MI_D2 vs. HFD groups, respectively (*p*.adj < 0.05, Tukey’s post-hoc test). HFD, high-fat diet. MWAT, mesenteric white adipose tissue. MUS, gastrocnemius and soleus muscles. CHOL, total cholesterol. TG, triacylglicerols. The body weights of the four groups at week 8 (just before the beginning of the placebo or CMA supplementation) were as follows (mean ± SD): Control: 123.05 ± 5.14; HFD: 121.07 ± 7.54; HFD + MI_D1: 119.85 ± 7.51; HFD + MI_D2: 122.55 ± 6.90.

## Data Availability

All raw RNA-sequencing data generated from this study can be accessed through accession number GSEXXXXXX.

## Supplementary Materials

The following are available online at https://www.mdpi.com/article/10.3390/biomedicines9101440/s1, Dataset S1: List of genes identified in liver transcriptomics and DEGs comparing HFD to control, HFD + MI_D1 to HFD, HFD + MI_D2 to HFD, and HFD + MI_D1 to HFD + MI_D2, respectively. Dataset S2: KEGG pathway and GO enrichment analysis for DEGs in HFD versus control and HFD + MI_D2 versus HFD. Dataset S3: Reporter metabolites are presented using the differential expression of genes in the liver tissue of the CMA-treated hamsters compared with that in the liver tissue of the HFD hamsters. Dataset S4: Composition of the diets used in the study.
